# Emerging Roles of Circular RNAs in Vascular Smooth Muscle Cell Dysfunction

**DOI:** 10.3389/fgene.2021.749296

**Published:** 2022-01-19

**Authors:** Zuo Pu, Jingbo Lu, Xiaohan Yang

**Affiliations:** Department of Cardiovascular Surgery, Fuwai Hospital Chinese Academy of Medical Sciences, Shenzhen, China

**Keywords:** circular RNAs, circRNAs, vascular smooth muscle cells, circ_0002579, circACTA2

## Abstract

Atherosclerosis is the major pathophysiological basis of cerebrovascular and cardiovascular diseases. Vascular smooth muscle cells (VSMCs) constitute the main structure of vasculature and play important roles in maintaining vascular tone and blood pressure. Many biological processes and cellular signaling events involved in atherosclerogenesis have been shown to converge on deregulating VSMC functions. However, the molecular mechanisms underlying dysfunctional VSMC in atherosclerosis are still poorly defined. Recent evidence revealed that circular RNAs (circRNAs) are closely related to diseases such as degenerative diseases, tumor, congenital diseases, endocrine diseases and cardiovascular diseases. Several studies demonstrated that circRNAs (e.g., circACTA2, Circ-SATB2, circDiaph3, circ_0020397, circTET3, circCCDC66) played critical roles in the regulation of VSMC proliferation, migration, invasion, and contractile-to-synthetic phenotype transformation by sponging microRNAs (e.g., miR-548f-5p, miR-939, miR-148a-5p, miR-138, miR-351-5p, miR-342-3p). This review describes recent progress in the profiling of circRNAs by transcriptome analysis in VSMCs and their molecular functions in regulating VSMC proliferation and migration.

## Introduction

Atherosclerosis is the major pathophysiological basis of cerebrovascular and cardiovascular diseases and can be attributed to the interactions of a myriad of risk factors (Wang et al., 2021a; Qi et al., 2021; Xuan et al., 2021). With the increasing ageing population in most developed countries, the mortality and morbidity of cardiovascular and cerebrovascular diseases are growing worldwide (Birger et al., 2021; Faggiano et al., 2021; Nasir and Cainzos-Achirica, 2021). Vascular smooth muscle cells (VSMCs) constitute the main structure of the vasculature and are key to the maintenance of vascular tone and blood pressure (Zhang et al., 2014; Cil et al., 2021; Zhu et al., 2021). VSMCs are maintained in the non-proliferative stage under the normal condition but can readily proliferate upon vascular injury (Lacolley et al., 2012; Kim and Kang, 2013; Olivieri et al., 2013). Increasing number of studies have indicated that abnormal migration and proliferation of VSMCs are common features of different vascular diseases, such as hypertension, vascular aneurysms, and atherosclerosis. In this regard, VSMCs are known to be heavily involved in atherosclerotic lesion formation (Liu et al., 2011; Yu et al., 2011; Gui et al., 2012; Xu et al., 2021). Many soluble factors and signaling pathways involved in atherosclerogenesis have been shown to deregulate VSMC migration and proliferation as well as transformation from the contractile to the synthetic phenotype (Song et al., 2012; Li et al., 2013; Blumensatt et al., 2014). However, the molecular mechanisms underlying VSMC dysfunctions are still poorly defined due to the complex interactions of VSMCs with their microenvironment and the heterogeneity of VSMCs (Shanahan and Weissberg, 1998; Kim et al., 2009). Therefore, it is pivotal to shed new light on the relevant cellular and molecular processes to develop mechanism-driven therapeutics for VSMC-related diseases.

Circular RNAs (CircRNAs) belong to a class of newly discovered endogenous regulatory RNAs that are generated by the formation of covalently closed loops that lack 3ʹ-poly-A tails and 5ʹ-caps through back-splicing. In this connection, circRNAs have shown high tissue stability, cross-species conversation, as well as disease stage- and tissue-specificity (Memczak et al., 2013; Ebbesen et al., 2016; Dai et al., 2019; Zhao et al., 2020a; Tu et al., 2020). Growing amount of evidence have demonstrated that most of the circRNAs function as competing endogenous RNAs to regulate gene expression post-transcriptionally *via* sponging microRNAs (miRNAs) (Li et al., 2015; Guo et al., 2020; Li et al., 2020; Ma et al., 2020). Some circRNAs could also bind to proteins directly to mediate their biological functions (Huang et al., 2020). It is noteworthy that, while majority of circRNAs are regarded as regulatory non-coding RNAs, a minor subset, particularly those having internal ribosome entry sites or N^6^-methyladenosine modification, could retain the ability to derive protein *via* a process known as rolling circle translation (Abe et al., 2015). Some circRNAs are even be localized in the nucleus to regulate transcription (Bose and Ain, 2018). Functionally, circRNAs are involved in the regulation in most, if not all, biological processes including cell differentiation, autophagy, apoptosis, invasion/migration, metabolism, and proliferation (Liu et al., 2019; Xu et al., 2019; Zheng et al., 2019; Pan et al., 2020; Wang et al., 2021b). It is therefore not surprising that deregulated expression of circRNAs is closely related to different types of diseases, such as degenerative diseases, tumor, congenital diseases, endocrine diseases and cardiovascular diseases (Lukiw, 2013; Li et al., 2019a; Chen et al., 2021a; Li et al., 2021; Papatsirou et al., 2021). With relevance to clinical practice, tissue circRNAs could act as potential biomarkers for prognostication and diagnosis of diseases, particularly tumors (Hu et al., 2019; Liu et al., 2020; Yao et al., 2020; Luo et al., 2021). Recently, several studies demonstrated that aberrant circRNA expression could contribute to the deregulated migration and proliferation of VSMCs (Qin et al., 2021).

In this review, we first summarize circRNA expression profiling studies in VSMCs to provide the scientific community with a comprehensive collection of datasets for selecting specific VSMC-associated circRNAs for further investigation in the future. Specific circRNAs with functional significance and their potential therapeutic exploitation will also be discussed.

## CircRNA Expression Profiling and Integrative Analysis in VSMCs

**FIGURE 1:**
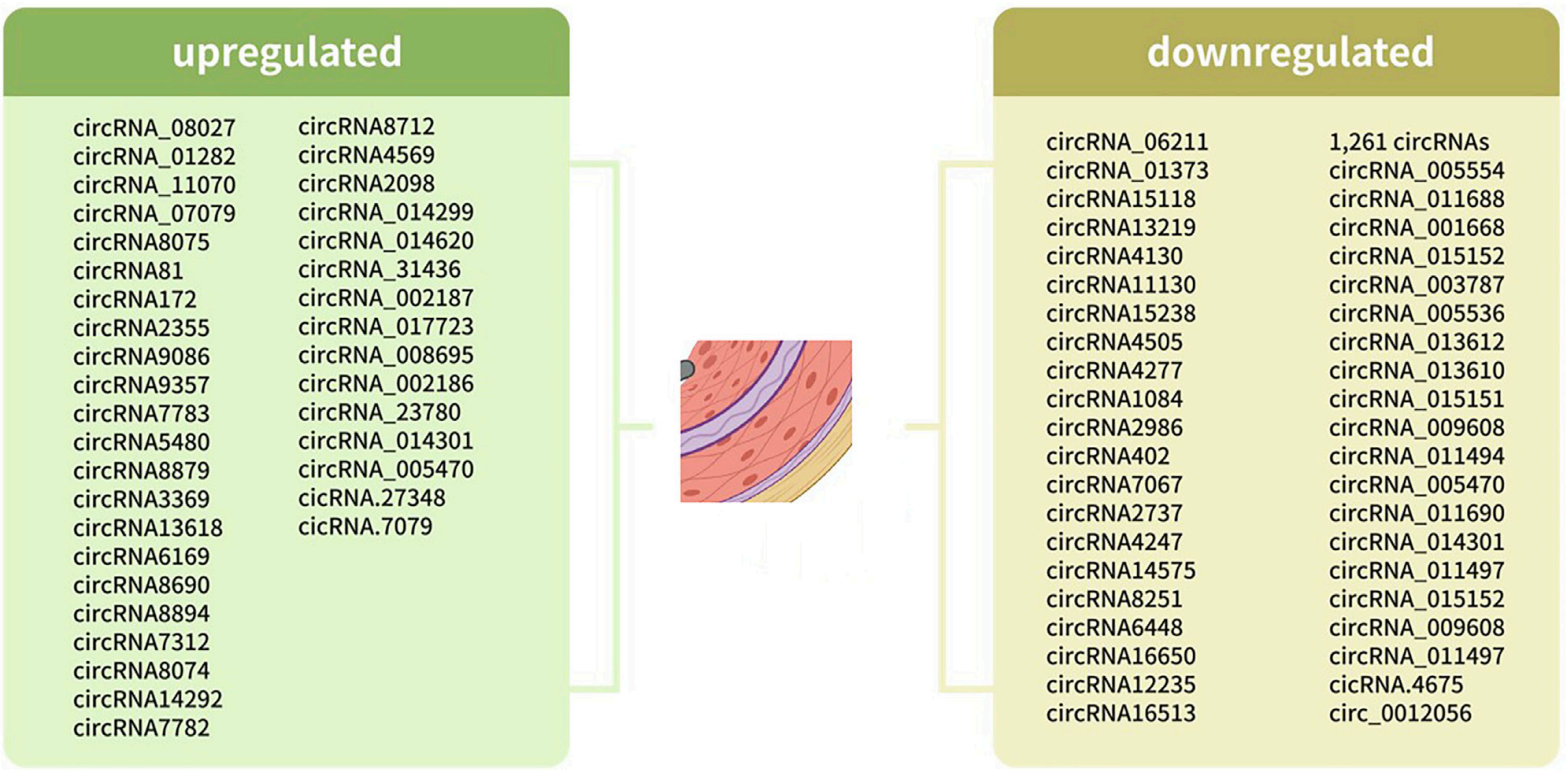
Differentially expressed circRNAs in different status of VSMCs.

Transcriptome-wide RNA sequencing technology has been used to identify the deregulated expression of non-coding regulatory RNAs, including miRNAs, long non-coding RNAs (lncRNAs) and circRNAs, through advanced sample processing workflows and newly developed computational algorithms. For shotgun sequencing-based circRNA profiling, the additional procedures usually include linear RNA removal through exonuclease digestion coupled with identification of back-spliced reads using specific bioinformatic programmes, such as CIRI2 (Gao et al., 2018), DCC (Cheng et al., 2016), Sailfish-cir (Li et al., 2017) and CIRIquant (Zhang et al., 2020), each of which has distinct sensitivity, reliability, and computational requirement. Aside from shotgun sequencing, a newer approach based on rolling circular reverse transcription and nanopore sequencing is available for the annotation of the full repertoires of circRNAs (Liu et al., 2021a). Microarrays with probes that target back-splice sites have also been widely used for circRNA profiling (Li et al., 2019b). In the next step, the identified deregulated circRNAs could be confirmed by RT-qPCR using divergent primers designed to span the circRNA backsplice junction sequence (Panda and Gorospe, 2018). In this respect, attempts have been performed to identify specific differentially expressed circRNAs in VSMCs of different conditions (Figure 1; Tables 1, 2).

**TABLE 1:** circRNAs expression profiles in vascular smooth muscle cells.

Num	Method	Sample	Upregulated	Downregulated	References
1	RNA sequencing	PDGF-BB-treated	59 circRNAs	53 circRNAs	Tian et al. (2020)
		VSMC			
2	Microarray	FBS-treated	66 CircRNAs	68 CircRNAs	Chen et al. (2020)
		VSMC			
3	Microarray	Common carotid artery	35 circRNAs	38 circRNAs	Hall et al. (2019)
4	RNA sequencing	Vein graft rat model	54 circRNAs	52 circRNAs	Liu et al. (2021b)
5	Microarray	PDGF-BB-treated	169 circRNAs	88 circRNAs	Peng et al. (2020)
		VSMC			
6	Microarray	Microarray-treated	44 circRNAs	3 circRNAs	Sun et al. (2020)
		VSMC			

**TABLE 2:** CircRNAs identified from RNA-sequencing or microarray were confirmed by RT-qPCR in vascular smooth muscle cells.

Num	Method	Sample	Upregulated	Downregulated	References
1	RNA-sequencing	PDGF-BB-treated	circRNA-14411, circRNA-13360, circRNA-4452, circRNA-8979 circRNA-1698	circRNA-5780, circRNA-3875, circRNA-3041 circRNA-1848	Tian et al. (2020)
	RT-PCR	VSMC			
2	Microarray RT-PCR	FBS-treated	circ_0002720, circ_0040705, circ_0009792, circ_0007422, circ_0001304 circ_0004872	circ_0057072, circ_0007146, circ_0009065, circ_0007888, circ_0006677 circ_0083756	Chen et al. (2020)
		VSMC			
3	RNA sequencing	Vein graft rat model	circ_0113656, circ_0001636 circ_00009732	—	Liu et al. (2021b)

Platelet-derived growth factor type BB (PDGF-BB) is known to induce VSMC dedifferentiation, migration and proliferation (Lu et al., 2018). Tian et al. used RNA-sequencing to profile circRNA expression of VSMCs exposed to PDGF-BB (Tian et al., 2020). VSMCs were treated without or with PDGF-BB (10 ng/ml). A total of 6,999 circRNAs were annotated, among which 94.06% were exonic, 5.43% were intronic and 0.50% were derived from intergenic regions. A total of 112 circRNAs were differentially expressed between the two VSMCs, with 53 circRNAs downregulated and 59 circRNAs upregulated in the PDGF-BB-treated group. The downregulation of circRNA-5780, circRNA-3875, circRNA-3041 and circRNA-1848 and the upregulation of circRNA-14411, circRNA-13360, circRNA-4452, circRNA-8979 and circRNA-1698 were confirmed using RT-qPCR. Furthermore, they showed that circ_0008776, which harbors 11 miRNA binding sites, had the highest degree of connectivity in the circRNA-miRNA network. In a similar study, Peng et al. used circRNA microarray to identify differentially expressed circRNAs in VSMCs upon exposure to PDGF-BB, in which 169 circRNAs were upregulated whereas 88 circRNAs were downregulated. qRT-PCR confirmed the overexpression of circ_0113656, circ_0001636 and circ_00009732 in the PDGF-BB group compared to control group.

Chen et al. used microarray to profile circRNA expression in quiescent and proliferative VSMCs cultured without or with fetal bovine serum, respectively (Chen et al., 2020). A total of 134 circRNAs were differentially expressed between the two groups, among which 66 circRNAs were upregulated and 68 circRNAs were downregulated in the proliferative group. These 134 circRNAs were divided into three types: 11% circRNAs were intronic, 5% circRNAs were intragenic and 84% circRNAs were exonic. The downregulation of circ_0057072, circ_0007146, circ_0009065, circ_0007888, circ_0006677 and circ_0083756 and the upregulation of circ_0002720, circ_0040705, circ_0009792, circ_0007422, circ_0001304 and circ_0004872 in the proliferative VSMCs were confirmed by RT-qPCR.

Xu et al. performed microarray to study circRNA expression in the balloon-mediated common carotid artery injury model (Hall et al., 2019). A total of 73 circRNAs were found to be differentially expressed, among which 35 circRNAs were upregulated and 38 circRNAs were downregulated in the balloon-injured common carotid artery.

Aberrant VSMC proliferation and migration to the intima contribute to vascular restenosis after the coronary artery bypass graft. Liu et al. utilized whole-transcriptome sequencing to identify differentially expressed circRNAs in an autologous vein graft model in rats (Liu et al., 2021b). 106 out of 2,048 annotated circRNAs were found to be deregulated, among which 54 circRNAs were upregulated and 52 circRNAs were downregulated.

Angiotensin II type 1 receptor (AT_1_R) autoantibody (AT_1_-AA) could contribute to vascular remodelling. Sun et al. used microarray to depict the circRNA expression landscape in AT_1_-AA-treated aortic smooth muscle cells (Sun et al., 2020). A total 47 circRNAs (44 upregulated and three downregulated) were differentially expressed were identified.

### Functionally Important circRNAs in VSMCs With Defined Mechanisms of Action

#### Circ_0002579

Chen et al. found that circ_0002579 was upregulated in the proliferative VSMCs as compared to quiescent VSMCs (Chen et al., 2020). Pathway and gene ontology analyses showed that circ_0002579 was co-expressed with 35 differentially expressed mRNAs that were enriched in the Ras, AMP-activated protein kinase (AMPK) and transforming growth factor (TGF)-β receptor signaling pathways. Circ_0,002,579 was predicted to sponge multiple miRNAs targeting high mobility group AT-hook 2 (HMGA2). Accordingly, knockdown of circ_0002579 downregulated HMGA2 protein level and reduced the expression of a proliferation marker (i.e., PCNA) in VSMCs.

#### CircACTA2

Sun et al. identified a new circRNA known as circACTA2 that was transcribed from exons five to nine of α-SMA (α-smooth muscle actin) gene. Functionally, circATCA2 sponges miR-548f-5p expression to promote the expression of α-SMA (Sun et al., 2017). Upstream, neuregulin-1 intracellular domain (NRG-1-ICD) was found to induce the expression of circACTA2. These data suggest that the NRG-1-ICD/circACTA2/miR-548f-5p/α-SMA axis may act as a novel treatment target for VSMC dysfunction. In another study, Ma et al. (Ma et al., 2021). demonstrated that circACTA2 was overexpressed in the vascular walls of hypertensive cases and in angiotensin II-induced VSMCs. Knockdown of circACTA2 inhibited angiotensin II-induced VSMC senescence as shown by inhibited expression of p21, enhanced expression of CDK4 and reduction of β-galactosidase-positive VSMCs. RNA immunoprecipitation and oligo pull-down assays demonstrated that both CDK4 mRNA and circACTA2 could bind to ILF3. Angiotensin II enhanced the interaction between circACTA2 and ILF3, thus releasing CDK4 mRNA which degraded rapidly in its unbound form. The authors’ data suggested that the ILF3-circACTA2-CDK4 axis may provide a new therapy target for ameliorating VSMC dysfunction in cardiovascular diseases.

#### Circ-SATB2

Mao et al. demonstrated that STIM1 and circ-SATB2 were overexpressed in the PDGF-BB-induced proliferative VSMCs, while the miR-939 level was downregulated. miR-939 and circ-SATB2 did not influence the level of each other but circ-SATB2 induced STIM1 expression whereas miR-939 suppressed STIM1 expression (Mao et al., 2018). Ectopic expression of circ-SATB2 also decreased SM22-alpha (SM22a) expression while SM22a level was enhanced *via* miR-939. Functionally, both circ-SATB2 and STIM1 induced cell migration and growth of VSMCs whereas overexpression of miR-939 suppressed VSMC migration and growth and induced apoptosis. Mechanistically, the modulatory effects of circ-SATB2 on VSMC apoptosis, migration, proliferation, and phenotypic differentiation were mediated through STIM1.

#### CircDiaph3

Xu et al. demonstrated that circDiaph3 was localized in the cell cytoplasm of VSMCs (Xu et al., 2019). Knockdown of circDiaph3 suppressed collagen I and cyclin D_1_ expression and inhibited VSMC migration and proliferation. Downregulation of circDiaph3 increased Diaph3 expression in VSMCs, in which miR-148a-5p may be one of the targets of circDiaph3. To this end, miR-148a-5p enhanced the expression of markers for contractile smooth muscle cells and suppressed VSMC migration and proliferation. Furthermore, they found that Igf1r was the direct target of miR-148a-5p and Igf1r level was upregulated in the balloon-injured common carotid artery. These data collectively showed that knockdown of circDiaph3 could suppress VSMC proliferation, migration, and dedifferentiation. This circRNA may be a new target for preventing intimal hyperplasia after vascular injury.

#### Circ_0020397

Wang et al. demonstrated that KDR and circ_0020397 were downregulated while miR-138 expression was upregulated in VSMC and arterial wall samples of intracranial aneurysm (Wang et al., 2019). Ectopic expression of circ_0,20397 induced VSMC growth whereas miR-138 induced VSMC apoptosis. Overexpression of circ_0020397 decreased miR-138 expression in VSMCs where these two non-coding RNAs were negatively correlated with each other. Moreover, KDR was found to be the target gene of miR-138. Overexpression of circ_0020397 induced VSMC growth *via* sponging the miR-138/KDR axis.

#### CircTET3

Yao et al. demonstrated that circTET3 was upregulated in the grafted vein as compared to the control (Yao et al., 2020). Knockdown of circTET3 suppressed migration of VSMCs where miR-351-5p was identified to be the direct target of circTET3. In contrast, ectopic expression of circTET3 promoted VSMC migration *via* sponging miR-351-5p. Their data suggested that the circTET3-miR-351-5p axis may be a novel potential treatment target for preventing intimal hyperplasia after vein graft.

#### CircCCDC66

CircCCDC66 was differentially expressed in abdominal aortic aneurysm. Yang et al. showed that depletion of circCCDC66 increased VSMC growth and reduced VSMC apoptosis (Yang et al., 2020). Mechanistically, circCCDC66 enhanced CCDC66 expression *via* sponging miR-342-3p to mediate its effect on VSMC proliferation and apoptosis. Their data suggested that the circCCDC66-miR-342-3p-CCDC66 axis plays a critical role in regulating VSMC function during abdominal aortic aneurysm.

#### CircCBFB

Yue et al. showed that circCBFB and miR-28-5p were enriched in the Ago2 protein isolated from VSMCs (Yue et al., 2020). Knockdown of circCBFB suppressed GRIA4 and LYPD3 expression, while knockdown of miR-28-5p revered these effects. Functionally, knockdown of circCBFB induced apoptosis of VSMCs, where LYPD3 and GRIA4 were inhibited by miR-28-5p. circCBFB acted as a sponge of miR-28-5p to release LYPD3 and GRIA4 from miR-28-5p-meidated inhibition. These signaling components were needed in circCBFB-regulated VSMC apoptosis. These data suggested that the circCBFB-miR-28-5p-GRIA4/LYPD3 axis is a key regulator of VSMC apoptosis.

#### Circ_Lrp6

Hall et al. identified a new circRNA, named circ_Lrp6, which was originated from the alternative splicing of lipoprotein receptor 6 (Lrp6), which was highly expressed in the vessels and involved in vascular pathologies (Hall et al., 2019). The authors showed that circ_Lrp6 sponged miR-145 expression as confirmed by luciferase assay and RNA immunoprecipitation. They also found that FASCIN, Yes1, KLF4, ITGβ8 and Lox were targets of miR-145 in VSMCs. Functionally, circ_Lrp6 dampened miR-145-regulated VSMC differentiation, growth, and migration. Knockdown of circ_Lrp6 inhibited intimal hyperplasia in the carotids. These data suggested that circ_Lrp6 is a potential target for preventing aberrant proliferation and migration of VSMCs.

#### CDR1as

Zhao et al. demonstrated that miR-7 expression was overexpressed, while the CKAP4 and CDR1as were decreased in the aortic samples from patients with abdominal aortic aneurysm as compared to the control group (Zhao et al., 2020b). Ectopic expression of CDR1as or knockdown of miR-7 enhanced VSMC growth whereas downregulation of CDR1as or overexpression of miR-7 produced the opposite effect. CKAP4 was found to be the direct target of miR-7.

#### CircErbB4

Sun et al. demonstrated that AT_1_-AA could induce migration of VSMCs *via* promoting the expression of angiotensin II type 2 receptor (AT_2_R). The authors also showed that circErbB4 (also known as circRNA-20314) was overexpressed in the AT_1_-AA-exposed mouse aortic smooth muscle cells (Sun et al., 2020). Mechanistically, AT_1_-AA increased the expression of circErbB4 and the RNA-binding protein Quaking (QKI) whose knockdown reduced circErbB4 formation. Overexpression of circErbB4 increased AT_2_R level whereas circErbB4 knockdown produced the opposite effect. The promoting effect of circErbB4 on AT_2_R was mediated through sponging miR-29a-5p. It was thus concluded that the QKI-circErbB4-AT_2_R axis plays a crucial role in AT_1_-AA-driven VSMC migration during vascular remodeling.

#### CircDHCR24

Peng et al. reported that circDHCR24 (also known as circ_0113,656) was upregulated in the PDGF-BB-exposed VSMCs (Peng et al., 2020). Knockdown of circDHCR24 suppressed VMSC migration and growth as well as enhancing the expression of two contractile markers (i.e., SM22α and α-SMA) expression but reduced the expression of a synthetic marker (i.e., osteopenia). Mechanistically, circDHCR24 disinhibited MMP9 *via* sponging miR-149-5p.

#### Circ_0,010,283

Ding et al. showed that HMGB1 and circ_0,010,283 levels were overexpressed in the oxidized low-density lipoprotein (ox-LDL)-exposed VSMCs in which miR-370-3p expression was decreased (Ding et al., 2020). Knockdown of circ_0,010,283 inhibited VSMC migration and growth and attenuated MMP2, MMP9 and cyclin D_1_ expression induced by ox-LDL. miR-370-3p was shown to be the target of circ_0,010,283 while HMGB1 was the direct target of miR-370-3p. circ_0010283 modulated HMGB1 expression through sponging miR-370-3p to mediate its effect on VMSC proliferation and migration. Ectopic expression of HMGB1 rescued the miR-370-3p-mediated inhibition of VSMC growth and migration. The authors’ data indicated that circ_0010283 promoted VSMC migration and growth *via* the miR-370-3p-HMGB1 axis in the ox-LDL-treated VSMCs.

#### CircSFMBT2

Luo and Chen showed that circSFMBT2 was upregulated in human neointimal samples obtained by atherectomy as compared to control samples and in PDGF-BB-treated VSMCs (Luo and Huang, 2021). Knockdown of circSFMBT2 suppressed VSMC migration and growth and enhanced the expression of contractile markers, namely, SMMHC, calponin and SM22α. Mechanistically, circSFMBT2 acted as a competing endogenous RNA to bind to miR-331-3p to derepress HDAC5, which decreased the transcription efficiency of Aggf1. These data indicated that circSFMBT2 is an important regulator of VSMC migration and growth *via* modulating the miR-331-3p-HDAC5-Aggf1 axis.

#### Circ_0020397

Yin et al. showed that circ_0020397 and GREM1 levels were downregulated in VSMCs isolated from patients with intracranial aneurysm (Yin and Liu, 2021). Ectopic expression of circ_0,020,397 or GREM1 induced VSMC proliferation whereas knockdown of circ_0020397 or GREM1 produced the opposite effect. Mechanistically, circ_0,020,397 was found to sponge miR-502-5p to promote GREM1 expression to mediate its promoting effect on VSMC proliferation.

#### CircUVRAG

Liu et al. used whole-transcriptome sequencing to show that circUVRAG was downregulated in the grafted vein (Liu et al., 2021b). Knockdown of circUVRAG inhibited VSMC migration and adhesion. Interestingly, the UVRAG pre-mRNA was found to be co-localized with NOVA1 in the nucleus while knockdown of NOVA1 inhibited the formation of both circUVRAG expression and linear UVRAG mRNA without altering the level of the UVRAG pre-mRNA. These data suggested that NOVA1 was involved in the modulation of formation of circUVRAG that can suppress VSMC migration and adhesion.

#### Circ-ARFIP2

Qin et al. showed that circ-ARFIP2 (circ_0,021,001, circRNA ADP ribosylation factor interacting protein 2) and KDR expression were downregulated whereas the miR-338-3p level was upregulated in the arterial wall samples isolated from patients with intracranial aneurysm (Qin et al., 2021). Ectopic expression of circ-ARFIP2 induced VSMCs migration, growth, and invasion partly *via* modulating miR-338-3p. In addition, they found that KDR was a target of miR-338-3p. Overexpression of circ-ARFIP2 enhanced KDR expression. Elevated expression of KDR also increased VSMC migration, growth, and invasion. Silencing of miR-338-3p produced the same effects *via* disinhibiting KDR expression. These data support that circ-ARFIP2 modulated KDR expression *via* sponging miR-338-3p.

#### Circ_CHFR

Wang et al. demonstrated that circ_CHFR was overexpressed in the PDGF-BB-treated VSMCs where knockdown of circ_CHFR decreased PDGF-BB-induced promotion of cell invasion, growth and migration and inhibition of apoptosis (Wang et al., 2021c). Mechanistically, circ_CHFR targeted miR-149-5p whose suppression attenuated the fucntional effects of circ_CHFR silencing in PDGF-BB-treated VSMCs. Furthermore, the authors showed that circ_CHFR enhanced NRP2 expression through sponging miR-149-5p. Overexpression of miR-149-5p abolished PDGF-BB-induced promotion of cell invasion, growth, and migration *via* regulating NRP2. These data suggested that PDGF-BB upregulated circ_CHFR to modulate the miR-149-5p-NRP2 axis to induce VSMC migration, growth, and invasion. Circ_CHFR may thus serve as a novel potential treatment target for inhibiting aberrant VSMC functions in atherosclerosis.

## Conclusion

Altered proliferation, migration, and contractile-to-synthetic phenotype transformation of VSMCs underlie the pathogenesis of many vascular diseases, such as hypertension, vascular aneurysms, and atherosclerosis. In this connection, a repertoire of circRNAs of functional significance to VSMCs (Table 3) have been identified by whole-transcriptome sequencing or circRNA microarray. These circRNAs mainly act as competing endogenous RNA to sponge miRNAs to derepress the downstream targets. The abovementioned studies also hinted at the potential clinical utility of targeting aberrantly upregulated circRNAs and their derepressed targets for therapeutic purpose. Silencing of these circRNAs with CRISPR/Cas9, antisense oligonucleotides or small interfering RNAs or blocking circRNA-miRNA interactions sterically by morpholinos for clinical translation in human are rapidly developing fields. Nevertheless, how to achieve tissue-specific delivery of these circRNA-targeting therapeutics remains a major technical hurdle. On the other hand, the research on the use of tissue or circulating circRNAs as biomarkers for predicting the progression of cardiovascular and cerebrovascular diseases is scarce. Future efforts should be put forth in this area. Finally, although many differentially expressed circRNAs have been identified by sequencing or microRNAs, the functions and mechanisms of action of only a handful of them have been appropriately studied, particularly in animals. In particular, vascular dysfunction is known to play a crucial regulatory role in tissue aging (Chen et al., 2021b; Chen et al., 2021c). How circRNA deregulation in VSMCs takes part in this process warrants further investigation. It is hopeful that further characterization of VSMC-related circRNAs will enhance our understanding of the pathogenesis of cardiovascular and cerebrovascular diseases and open up novel therapeutic avenue.

**TABLE 3:** Differentially expressed circRNAs in vascular smooth muscle cells of different phenotypes.

Name	Dysregulation	Sponge target	Phenotype(s) altered by the circRNA	Related gene	Role	References
Circ_0002579	Upregulated	—	Proliferation	HMGA2	Harmful	Chen et al. (2020)
circACTA2	Upregulated	miR-548f-5p	Contractile-to-synthetic phenotype transformation	α-SMA	Harmful	Sun et al. (2017)
				NRG-1-ICD		
circACTA2	Upregulated	—	Senescence	CDK4	Harmful	Ma et al. (2021)
				ILF3		
Circ-SATB2	Upregulated	miR-939	Migaration apoptosis proliferation	STIM1	Harmful	Mao et al. (2018)
			Differentiation	SM22a		
circDiaph3	Upregulated	miR-148a-5p	Migration Proliferation dedifferentiation	collagen I	Harmful	Xu et al. (2019)
				cyclinD1		
				Diaph3		
				Igf1r		
circ_0,020,397	Downregulated	miR-138	Apoptosis proliferation	KDR	Protective	Wang et al. (2019)
circTET3	Upregulated	miR-351-5p	Migration	—	Harmful	Yao et al. (2020)
circCCDC66	Upregulated	miR-342-3p	Apoptosis proliferation	CCDC66	Harmful	Yang et al. (2020)
CircCBFB	Upregulated	miR-28-5p	Apoptosis	GRIA4	Harmful	Yue et al. (2020)
				LYPD3		
Circ_Lrp6	Upregulated	miR-145	Differentiation Migration proliferation	FASCIN, Yes1	Harmful	Hall et al. (2019)
				KLF4		
				ITGβ8		
				Lox		
CDR1as	Downregulated	miR-7	Proliferation apoptosis	LDH	Protective	Zhao et al. (2020b)
				ROS		
				CKAP4		
circErbB4	Upregulated	miR-29a-5p	Migration	AT2R	Harmful	Sun et al. (2020)
				QKI		
				ADAR1		
circDHCR24	Upregulated	miR-149-5p	Migration	MMP9	Harmful	Peng et al. (2020)
			Proliferation contractile			
circ_0,010,283	Upregulated	miR-370-3p	Migration	HMGB1	Harmful	Ding et al. (2020)
			Proliferation			
CircSFMBT2	Upregulated	miR-331	Migration	Aggf1	Harmful	Luo and Huang, (2021)
			Proliferation	HDAC5		
Circ_0,020,397	Downregulated	miR-502-5p	Proliferation	GREM1	Protective	Yin and Liu, (2021)
circUVRAG	Downregulated	—	Migration adhesion	NOVA1	Protective	Liu et al. (2021b)
circ-ARFIP2	Downregulated	miR-338-3p	Migration, proliferation invasion	KDR	Protective	Qin et al. (2021)
Circ_CHFR	Upregulated	miR-149-5p	Iinvasion, proliferation migration	NRP2	Harmful	Wang et al. (2021c)

## References

[B1] Abe N. Matsumoto K. Nishihara M. Nakano Y. Shibata A. Maruyama H. (2015). Rolling Circle Translation of Circular RNA in Living Human Cells. Sci. Rep. 5, 16435. 10.1038/srep16435 26553571 PMC4639774

[B2] Birger M. Kaldjian A. S. Roth G. A. Moran A. E. Dieleman J. L. Bellows B. K. (2021). Spending on Cardiovascular Disease and Cardiovascular Risk Factors in the United States: 1996 - 2016. Circulation 144, 271–282. 10.1161/circulationaha.120.053216 33926203 PMC8316421

[B3] Blumensatt M. Wronkowitz N. Wiza C. Cramer A. Mueller H. Rabelink M. J. (2014). Adipocyte-derived Factors Impair Insulin Signaling in Differentiated Human Vascular Smooth Muscle Cells via the Upregulation of miR-143. Biochim. Biophys. Acta (Bba) - Mol. Basis Dis. 1842 (2), 275–283. 10.1016/j.bbadis.2013.12.001 24333576

[B4] Bose R. Ain R. (2018). Regulation of Transcription by Circular RNAs. Adv. Exp. Med. Biol. 1087, 81–94. 10.1007/978-981-13-1426-1_7 30259359

[B5] Chen H. Zhang S. Wu Y. Li Z. Wang D. Cai S. (2021). The Role of Circular RNA Circ_0008285 in Gestational Diabetes Mellitus by Regulating the Biological Functions of Trophoblasts. Biol. Res. 54 (1), 14. 10.1186/s40659-021-00337-3 33879262 PMC8056579

[B6] Chen J. Lippo L. Labella R. Tan S. L. Marsden B. D. Dustin M. L. (2021). Decreased Blood Vessel Density and Endothelial Cell Subset Dynamics during Ageing of the Endocrine System. EMBO J. 40 (1), e105242. 10.15252/embj.2020105242 33215738 PMC7780152

[B7] Chen J. Sivan U. Tan S. L. Lippo L. De Angelis J. Labella R. (2021). High-resolution 3D Imaging Uncovers Organ-specific Vascular Control of Tissue Aging. Sci. Adv. 7 (6), 1. 10.1126/sciadv.abd7819 PMC7857692 33536212

[B8] Chen W. Lin J. Li B. Cao S. Li H. Zhao J. (2020). Screening and Functional Prediction of Differentially Expressed circRNAs in Proliferative Human Aortic Smooth Muscle Cells. J. Cel Mol Med 24 (8), 4762–4772. 10.1111/jcmm.15150 PMC7176856 32155686

[B9] Cheng J. Metge F. Dieterich C. (2016). Specific Identification and Quantification of Circular RNAs from Sequencing Data. Bioinformatics 32 (7), 1094–1096. 10.1093/bioinformatics/btv656 26556385

[B10] Cil O. Chen X. Askew Page H. R. Baldwin S. N. Jordan M. C. Myat Thwe P. (2021). A Small Molecule Inhibitor of the Chloride Channel TMEM16A Blocks Vascular Smooth Muscle Contraction and Lowers Blood Pressure in Spontaneously Hypertensive Rats. Kidney Int. 100, 311–320. 10.1016/j.kint.2021.03.025 33836171 PMC8319106

[B11] Dai X. Guo X. Liu J. Cheng A. Peng X. Zha L. (2019). Circular RNA circGRAMD1B Inhibits Gastric Cancer Progression by Sponging miR-130a-3p and Regulating PTEN and P21 Expression. Aging 11 (21), 9689–9708. 10.18632/aging.102414 31719211 PMC6874462

[B12] Ding P. Ding Y. Tian Y. Lei X. (2020). Circular RNA Circ_0010283 Regulates the Viability and Migration of Oxidized Low-density L-ipoprotein-induced V-ascular S-mooth M-uscle C-ells via an miR-370-3p/HMGB1 axis in A-therosclerosis. Int. J. Mol. Med. 46 (4), 1399–1408. 10.3892/ijmm.2020.4703 32945389 PMC7447304

[B13] Ebbesen K. K. Kjems J. Hansen T. B. (2016). Circular RNAs: Identification, Biogenesis and Function. Biochim. Biophys. Acta (Bba) - Gene Regul. Mech. 1859, 163–168. 10.1016/j.bbagrm.2015.07.007 26171810

[B14] Faggiano A. Santangelo G. Carugo S. Pressman G. Picano E. Faggiano P. (2021). Cardiovascular Calcification as a Marker of Increased Cardiovascular Risk and a Surrogate for Subclinical Atherosclerosis: Role of Echocardiography. Jcm 10 (8), 1668. 10.3390/jcm10081668 33924667 PMC8069968

[B15] Gao Y. Zhang J. Zhao F. (2018). Circular RNA Identification Based on Multiple Seed Matching. Brief Bioinform 19 (5), 803–810. 10.1093/bib/bbx014 28334140

[B16] Gui T. Zhou G. Sun Y. Shimokado A. Itoh S. Oikawa K. (2012). MicroRNAs that Target Ca2+ Transporters Are Involved in Vascular Smooth Muscle Cell Calcification. Lab. Invest. 92 (9), 1250–1259. 10.1038/labinvest.2012.85 22688076

[B17] Guo F. Li S. Guo C. Xu X. Zhou X. Ma D. (2020). Circular RNA circMAGI3 Accelerates the Glycolysis of Non-small Cell Lung Cancer through miR-515-5p/HDGF. Am. J. Transl Res. 12 (7), 3953–3963. 32774748 PMC7407684

[B18] Hall I. F. Climent M. Quintavalle M. Farina F. M. Schorn T. Zani S. (2019). Circ_Lrp6, a Circular RNA Enriched in Vascular Smooth Muscle Cells, Acts as a Sponge Regulating miRNA-145 Function. Circ. Res. 124 (4), 498–510. 10.1161/circresaha.118.314240 30582454

[B19] Hu C. Wang Y. Li A. Zhang J. Xue F. Zhu L. (2019). Overexpressed Circ_0067934 Acts as an Oncogene to Facilitate Cervical Cancer Progression via the miR-545/EIF3C axis. J. Cel Physiol 234 (6), 9225–9232. 10.1002/jcp.27601 30362562

[B20] Huang A. Zheng H. Wu Z. Chen M. Huang Y. (2020). Circular RNA-Protein Interactions: Functions, Mechanisms, and Identification. Theranostics 10 (8), 3503–3517. 10.7150/thno.42174 32206104 PMC7069073

[B21] Kim S. Kang H. (2013). miR-15b Induced by Platelet-Derived Growth Factor Signaling Is Required for Vascular Smooth Muscle Cell Proliferation. BMB Rep. 46 (11), 550–554. 10.5483/bmbrep.2013.46.11.057 24152911 PMC4133843

[B22] Kim Y. S. Galis Z. S. Rachev A. Han H.-C. Vito R. P. (2009). Matrix Metalloproteinase-2 and -9 Are Associated with High Stresses Predicted Using a Nonlinear Heterogeneous Model of Arteries. J. biomechanical Eng. 131 (1), 011009. 10.1115/1.3005163 PMC8455176 19045925

[B23] Lacolley P. Regnault V. Nicoletti A. Li Z. Michel J.-B. (2012). The Vascular Smooth Muscle Cell in Arterial Pathology: a Cell that Can Take on Multiple Roles. Cardiovasc. Res. 95 (2), 194–204. 10.1093/cvr/cvs135 22467316

[B24] Li J. Yang J. Zhou P. Le Y. Zhou C. Wang S. (2015). Circular RNAs in Cancer: Novel Insights into Origins, Properties, Functions and Implications. Am. J. Cancer Res. 5 (2), 472–480. 25973291 PMC4396047

[B25] Li M. Xie X. Zhou J. Sheng M. Yin X. Ko E.-A. (2017). Quantifying Circular RNA Expression from RNA-Seq Data Using Model-Based Framework. Bioinformatics 33 (14), 2131–2139. 10.1093/bioinformatics/btx129 28334396

[B26] Li P. Liu Y. Yi B. Wang G. You X. Zhao X. (2013). MicroRNA-638 Is Highly Expressed in Human Vascular Smooth Muscle Cells and Inhibits PDGF-BB-Induced Cell Proliferation and Migration through Targeting Orphan Nuclear Receptor NOR1. Cardiovasc. Res. 99 (1), 185–193. 10.1093/cvr/cvt082 23554459 PMC3687750

[B27] Li S. Teng S. Xu J. Su G. Zhang Y. Zhao J. (2019). Microarray Is an Efficient Tool for circRNA Profiling. Brief Bioinform 20 (4), 1420–1433. 10.1093/bib/bby006 29415187

[B28] Li Z. Chen Z. Feng Y. Hu G. Jiang Y. (2020). CircMMP11 Acts as a Ce-circRNA in Breast Cancer Progression by Regulating miR-1204. Am. J. Transl Res. 12 (6), 2585–2599. 32655792 PMC7344057

[B29] Li Z. Chen X. Xu D. Li S. Chan M. T. V. Wu W. K. K. (2019). Circular RNAs in Nucleus Pulposus Cell Function and Intervertebral Disc Degeneration. Cell Prolif 52 (6), e12704. 10.1111/cpr.12704 31621141 PMC6869348

[B30] Li Z. Li X. Xu D. Chen X. Li S. Zhang L. (2021). An Update on the Roles of Circular RNAs in Osteosarcoma. Cel Prolif 54 (1), e12936. 10.1111/cpr.12936 PMC7791175 33103338

[B31] Liu P. Li X. Guo X. Chen J. Li C. Chen M. (2019). Circular RNA DOCK1 Promotes Bladder Carcinoma Progression via Modulating circDOCK1/hsa-miR-132-3p/Sox5 Signalling Pathway. Cel Prolif 52 (4), e12614. 10.1111/cpr.12614 PMC6668968 30983072

[B32] Liu Q. Wang C. Jiang Z. Li S. Li F. Tan H. B. (2020). circRNA 001306 Enhances Hepatocellular Carcinoma Growth by Up‐regulating CDK16 Expression via Sponging miR‐584‐5p. J. Cel. Mol. Med. 24, 14306–14315. 10.1111/jcmm.16047 PMC7754030 33135290

[B33] Liu X. Cheng Y. Chen X. Yang J. Xu L. Zhang C. (2011). MicroRNA-31 Regulated by the Extracellular Regulated Kinase Is Involved in Vascular Smooth Muscle Cell Growth via Large Tumor Suppressor Homolog 2. J. Biol. Chem. 286 (49), 42371–42380. 10.1074/jbc.M111.261065 22020941 PMC3234904

[B34] Liu Z. Lou Y. Cui J.-C. Chen Y. Liu J.-T. Yuan Y. (2021). Circular RNA UVRAG Mediated by Alternative Splicing Factor NOVA1 Regulates Adhesion and Migration of Vascular Smooth Muscle Cells. Genes 12 (3), 418. 10.3390/genes12030418 33799408 PMC7999860

[B35] Liu Z. Tao C. Li S. Du M. Bai Y. Hu X. (2021). circFL-Seq Reveals Full-Length Circular RNAs with Rolling Circular Reverse Transcription and Nanopore Sequencing. Elife 10. 10.7554/eLife.69457 PMC8550772 34647522

[B36] Lu Q.-B. Wan M.-Y. Wang P.-Y. Zhang C.-X. Xu D.-Y. Liao X. (2018). Chicoric Acid Prevents PDGF-BB-Induced VSMC Dedifferentiation, Proliferation and Migration by Suppressing ROS/NFκB/mTOR/P70S6K Signaling cascade. Redox Biol. 14, 656–668. 10.1016/j.redox.2017.11.012 29175753 PMC5716955

[B37] Lukiw W. J. (2013). Circular RNA (circRNA) in Alzheimer's Disease (AD). Front. Genet. 4, 307. 10.3389/fgene.2013.00307 24427167 PMC3875874

[B38] Luo Y. Huang C. (2021). CircSFMBT2 Facilitates Vascular Smooth Muscle Cell Proliferation by Targeting miR-331-3p/HDAC5. Life Sci. 264, 118691. 10.1016/j.lfs.2020.118691 33166591

[B39] Luo Z. Lu L. Tang Q. Wei W. Chen P. Chen Y. (2021). CircCAMSAP1 Promotes Hepatocellular Carcinoma Progression through miR‐1294/GRAMD1A Pathway. J. Cel Mol Med 25, 3793–3802. 10.1111/jcmm.16254 PMC8051675 33484498

[B40] Ma D. Liu H. Qin Y. Li D. Cui Y. Li L. (2020). Circ_0007142/miR-186/FOXK1 axis Promoted Lung Adenocarcinoma Progression. Am. J. Transl Res. 12 (8), 4728–4738. 32913545 PMC7476148

[B41] Ma Y. Zheng B. Zhang X.-H. Nie Z.-Y. Yu J. Zhang H. (2021). circACTA2 Mediates Ang II-Induced VSMC Senescence by Modulation of the Interaction of ILF3 with CDK4 mRNA. Aging 13 (8), 11610–11628. 10.18632/aging.202855 33885378 PMC8109074

[B42] Mao Y.-y. Wang J.-q. Guo X.-x. Bi Y. Wang C.-x. (2018). Circ-SATB2 Upregulates STIM1 Expression and Regulates Vascular Smooth Muscle Cell Proliferation and Differentiation through miR-939. Biochem. Biophysical Res. Commun. 505 (1), 119–125. 10.1016/j.bbrc.2018.09.069 30241943

[B43] Memczak S. Jens M. Elefsinioti A. Torti F. Krueger J. Rybak A. (2013). Circular RNAs Are a Large Class of Animal RNAs with Regulatory Potency. Nature 495 (7441), 333–338. 10.1038/nature11928 23446348

[B44] Nasir K. Cainzos-Achirica M. (2021). Role of Coronary Artery Calcium Score in the Primary Prevention of Cardiovascular Disease. Bmj 373, n776. 10.1136/bmj.n776 33947652

[B45] Olivieri F. Recchioni R. Marcheselli F. Marie Abbatecola A. Santini G. Borghetti G. (2013). Cellular Senescence in Cardiovascular Diseases: Potential Age-Related Mechanisms and Implications for Treatment. Cpd 19 (9), 1710–1719. 10.2174/1381612811319090018 23061728

[B46] Pan G. Mao A. Liu J. Lu J. Ding J. Liu W. (2020). Circular RNA Hsa_circ_0061825 (circ‐TFF1) Contributes to Breast Cancer Progression through Targeting miR‐326/TFF1 Signalling. Cel Prolif 53 (2), e12720. 10.1111/cpr.12720 PMC7048212 31961997

[B47] Panda A. Gorospe M. (2018). Detection and Analysis of Circular RNAs by RT-PCR. Bio-protocol 8 (6). 10.21769/BioProtoc.2775 PMC5891140 29644261

[B48] Papatsirou M. Artemaki P. I. Karousi P. Scorilas A. Kontos C. K. (2021). Circular RNAs: Emerging Regulators of the Major Signaling Pathways Involved in Cancer Progression. Cancers 13 (11), 2744. 10.3390/cancers13112744 34205978 PMC8198587

[B49] Peng W. Li T. Pi S. Huang L. Liu Y. (2020). Suppression of Circular RNA circDHCR24 Alleviates Aortic Smooth Muscle Cell Proliferation and Migration by Targeting miR-149-5p/MMP9 axis. Biochem. Biophysical Res. Commun. 529 (3), 753–759. 10.1016/j.bbrc.2020.06.067 32736703

[B50] Qi P. Feng X. Lu J. Wang J. Hu S. Wang D. (2021). Morphological Irregularity of Unruptured Intracranial Aneurysms Is More Related with Aneurysm Size rather Than Cerebrovascular Atherosclerosis: A Case-Control Study. Cia 16, 665–674. 10.2147/cia.s301326 PMC8069126 33907388

[B51] Qin K. Tian G. Zhou D. Chen G. (2021). Circular RNA Circ-ARFIP2 Regulates Proliferation, Migration and Invasion in Human Vascular Smooth Muscle Cells via miR-338-3p-dependent Modulation of KDR. Metab. Brain Dis. 36, 1277–1288. 10.1007/s11011-021-00726-3 33837886

[B52] Shanahan C. M. Weissberg P. L. (1998). Smooth Muscle Cell Heterogeneity. Atvb 18 (3), 333–338. 10.1161/01.atv.18.3.333 9514400

[B53] Song L. Duan P. Guo P. Li D. Li S. Xu Y. (2012). Downregulation of miR-223 and miR-153 Mediates Mechanical Stretch-Stimulated Proliferation of Venous Smooth Muscle Cells via Activation of the Insulin-like Growth Factor-1 Receptor. Arch. Biochem. Biophys. 528 (2), 204–211. 10.1016/j.abb.2012.08.015 23046980

[B54] Sun Y. Li Y. Wang M. Yue M. Bai L. Bian J. (2020). Increased AT2R Expression Is Induced by AT1R Autoantibody via Two Axes, Klf-5/IRF-1 and circErbB4/miR-29a-5p, to Promote VSMC Migration. Cell Death Dis 11 (6), 432. 10.1038/s41419-020-2643-5 32514012 PMC7280191

[B55] Sun Y. Yang Z. Zheng B. Zhang X.-h. Zhang M.-l. Zhao X.-s. (2017). A Novel Regulatory Mechanism of Smooth Muscle α-Actin Expression by NRG-1/circACTA2/miR-548f-5p Axis. Circ. Res. 121 (6), 628–635. 10.1161/circresaha.117.311441 28698179

[B56] Tian J. Fu Y. Li Q. Xu Y. Xi X. Zheng Y. (2020). Differential Expression and Bioinformatics Analysis of CircRNA in PDGF-BB-Induced Vascular Smooth Muscle Cells. Front. Genet. 11, 530. 10.3389/fgene.2020.00530 32547599 PMC7272660

[B57] Tu F. L. Guo X. Q. Wu H. X. He Z. Y. Wang F. Sun A. J. (2020). Circ-0001313/miRNA-510-5p/AKT2 axis Promotes the Development and Progression of colon Cancer. Am. J. Transl Res. 12 (1), 281–291. undefined. 32051753 PMC7013220

[B58] Wang B. Zhao Z. Liu S. Wang S. Chen Y. Xu Y. (2021). Impact of Diabetes on Subclinical Atherosclerosis and Major Cardiovascular Events in Individuals with and without Non-alcoholic Fatty Liver Disease. Diabetes Res. Clin. Pract. 177, 108873. 10.1016/j.diabres.2021.108873 34051282

[B59] Wang L. Li B. Yi X. Xiao X. Zheng Q. Ma L. (2021). Circ_SMAD4 Promotes Gastric Carcinogenesis by Activating Wnt/β‐catenin Pathway. Cel Prolif 54 (3), e12981. 10.1111/cpr.12981 PMC7941240 33458917

[B60] Wang M. Li C. Cai T. Zhang A. Cao J. Xin H. (2021). Circ_CHFR Promotes PDGF-BB-Induced Proliferation, Invasion and Migration in VSMCs via miR-149-5p/NRP2 axis. J. Cardiovasc. Pharmacol. 1, 1. 10.1097/fjc.0000000000001055 33990513

[B61] Wang Y. Wang Y. Li Y. Wang B. Liu Z. Ma Y. (2019). Decreased Expression of Circ_0020397 in Intracranial Aneurysms May Be Contributing to Decreased Vascular Smooth Muscle Cell Proliferation via Increased Expression of miR-138 and Subsequent Decreased KDR Expression. Cell Adhes. Migration 13 (1), 219–227. 10.1080/19336918.2019.1619432 PMC6550538 31096819

[B62] Xu J.-Y. Chang N.-B. Rong Z.-H. Li T. Xiao L. Yao Q.-P. (2019). circDiaph3 Regulates Rat Vascular Smooth Muscle Cell Differentiation, Proliferation, and Migration. FASEB j. 33 (2), 2659–2668. 10.1096/fj.201800243RRR 30307766

[B63] Xu T. Jia J. Xu N. Ye C. Zheng F. Yuan Y. (2021). Apelin Receptor Upregulation in Spontaneously Hypertensive Rat Contributes to the Enhanced Vascular Smooth Muscle Cell Proliferation by Activating Autophagy. Ann. Transl Med. 9 (8), 627. 10.21037/atm-20-6891 33987325 PMC8106044

[B64] Xuan J. Shang M. Li X. (2021). Serum MicroRNA-137 Serves as a Novel Biomarker for Cerebral Atherosclerosis Diagnosis and Cerebrovascular Event Prediction. J. Cardiovasc. Pharmacol. 78, 302–307. 10.1097/fjc.0000000000001058 34050091 PMC8340946

[B65] Yang R. Wang Z. Meng G. Hua L. (2020). Circular RNA CCDC66 Facilitates Abdominal Aortic Aneurysm through the Overexpression of CCDC66. Cell Biochem Funct 38 (7), 830–838. 10.1002/cbf.3494 31997404

[B66] Yao Q. P. Liu Z. Yao A. H. Liu J. T. Jiang J. Chen Y. (2020). Circular RNA circTET3 Mediates Migration of Rat Vascular Smooth Muscle Cells by Targeting miR‐351‐5p. J. Cel Physiol 235 (10), 6831–6842. 10.1002/jcp.29577 31990052

[B67] Yin K. Liu X. (2021). Circ_0020397 Regulates the Viability of Vascular Smooth Muscle Cells by Up-Regulating GREM1 Expression via miR-502-5p in Intracranial Aneurysm. Life Sci. 265, 118800. 10.1016/j.lfs.2020.118800 33242525

[B68] Yu M.-L. Wang J.-F. Wang G.-K. You X.-H. Zhao X.-X. Jing Q. (2011). Vascular Smooth Muscle Cell Proliferation Is Influenced by Let-7d microRNA and its Interaction with KRAS. Circ. J. 75 (3), 703–709. 10.1253/circj.cj-10-0393 21266788

[B69] Yue J. Zhu T. Yang J. Si Y. Xu X. Fang Y. (2020). CircCBFB-mediated miR-28-5p Facilitates Abdominal Aortic Aneurysm via LYPD3 and GRIA4. Life Sci. 253, 117533. 10.1016/j.lfs.2020.117533 32151690

[B70] Zhang J. Chen S. Yang J. Zhao F. (2020). Accurate Quantification of Circular RNAs Identifies Extensive Circular Isoform Switching Events. Nat. Commun. 11 (1), 90. 10.1038/s41467-019-13840-9 31900416 PMC6941955

[B71] Zhang L. Zhou M. Wang Y. Huang W. Qin G. Weintraub N. L. (2014). miR-92a Inhibits Vascular Smooth Muscle Cell Apoptosis: Role of the MKK4-JNK Pathway. Apoptosis 19 (6), 975–983. 10.1007/s10495-014-0987-y 24705900 PMC4143895

[B72] Zhao D. Liu H. Liu H. Zhang X. Zhang M. Kolluri V. K. (2020). Downregulated Expression of Hsa_circ_0037515 and Hsa_circ_0037516 as Novel Biomarkers for Non-small Cell Lung Cancer. Am. J. Transl Res. 12 (1), 162–170. undefined. 32051745 PMC7013216

[B73] Zhao F. Chen T. Jiang N. (2020). CDR1as/miR-7/CKAP4 axis C-ontributes to the P-athogenesis of A-bdominal A-ortic A-neurysm by R-egulating the P-roliferation and A-poptosis of P-rimary V-ascular S-mooth M-uscle C-ells. Exp. Ther. Med. 19 (6), 3760–3766. 10.3892/etm.2020.8622 32346440 PMC7185088

[B74] Zheng S. Qian Z. Jiang F. Ge D. Tang J. Chen H. (2019). CircRNA LRP6 Promotes the Development of Osteosarcoma via Negatively Regulating KLF2 and APC Levels. Am. J. Transl Res. 11 (7), 4126–4138. 31396323 PMC6684910

[B75] Zhu G.-X. Zuo J.-L. Xu L. Li S.-Q. (2021). Ginsenosides in Vascular Remodeling: Cellular and Molecular Mechanisms of Their Therapeutic Action. Pharmacol. Res. 169, 105647. 10.1016/j.phrs.2021.105647 33964471

